# Thixotropic Hydrogels Composed of Self-Assembled Nanofibers of Double-Hydrophobic Elastin-Like Block Polypeptides

**DOI:** 10.3390/ijms22084104

**Published:** 2021-04-15

**Authors:** Yusuke Sugioka, Jin Nakamura, Chikara Ohtsuki, Ayae Sugawara-Narutaki

**Affiliations:** 1 Department of Materials Chemistry, Graduate School of Engineering, Nagoya University, Furo-cho, Chikusa-ku, Nagoya 464-8603, Japan; yusuke.s.es@gmail.com (Y.S.); nakamura@chembio.nagoya-u.ac.jp (J.N.); ohtsuki@chembio.nagoya-u.ac.jp (C.O.); 2 Department of Energy Engineering, Graduate School of Engineering, Nagoya University, Furo-cho, Chikusa-ku, Nagoya 464-8603, Japan

**Keywords:** elastin-like polypeptide, physical hydrogel, thixotropic gel, self-healing, chemical crosslinking

## Abstract

Physically crosslinked hydrogels with thixotropic properties attract considerable attention in the biomedical research field because their self-healing nature is useful in cell encapsulation, as injectable gels, and as bioinks for three-dimensional (3D) bioprinting. Here, we report the formation of thixotropic hydrogels containing nanofibers of double-hydrophobic elastin-like polypeptides (ELPs). The hydrogels are obtained with the double-hydrophobic ELPs at 0.5 wt%, the concentration of which is an order of magnitude lower than those for previously reported ELP hydrogels. Although the kinetics of hydrogel formation is slower for the double-hydrophobic ELP with a cell-binding sequence, the storage moduli G′ of mature hydrogels are similar regardless of the presence of a cell-binding sequence. Reversible gel–sol transitions are demonstrated in step-strain rheological measurements. The degree of recovery of the storage modulus G′ after the removal of high shear stress is improved by chemical crosslinking of nanofibers when intermolecular crosslinking is successful. This work would provide deeper insight into the structure–property relationships of the self-assembling polypeptides and a better design strategy for hydrogels with desired viscoelastic properties.

## 1. Introduction

Hydrogels have attracted great attention as three-dimensional (3D) cell culture scaffolds since they act like an in vivo microenvironment around cells [1,2,3]. It is important to design hydrogels with proper physicochemical and mechanical properties, especially in tissue regeneration and tissue model construction because cellular behaviors are siginificantly affected by those properties. In these contexts, biomimicry of the natural extracellular matrix (ECM) is a rational approach to constructing successful hydrogels for tissue regeneration.

The principal component of ECM is collagen, and its extraction, purification, and processing from animals have been established. Various types of collagen gels are available, and they are widely used in tissue regeneration. Elastin is another essential ECM protein in soft tissues, such as blood vessels, lungs, ligaments, and skin [4]. The mechanical properties of these tissues are controlled by a combination of collagen and elastin. Collagen provides tensile strength, while elastin provides extensibility and reversible recoil [5]. Unlike collagen, however, the use of naturally occurring elastin as cell culture scaffolds is underexplored because of the difficulty of purifying and processing elastin, caused by its intrinsic insolubility.

Researchers have attempted to develop elastin-like hydrogels using the biomimicry approach [6,7]. Chemically crosslinked, cell-compatible elastin-like hydrogels have been constructed carefully designing the crosslinking reactions. Weiss et al. synthesized methacrylate derivative of tropoelastin, the monomeric form of elastin [8,9]. This derivative is photo-crosslinked to form hydrogels within a minute upon ultraviolet (UV) irradiation. Because the period of UV irradiation is short enough, the process is compatible with cell encapsulation. Elastin-like polypeptides (ELPs) are a class of polypeptides that consists of repetitive amino acid motifs of natural elastin [10]. Typical ELPs consist of repeats of Val-Pro-Gly-Xaa-Gly (VPGXG), where Xaa is a guest amino acid residue, except for Pro. By incorporating Lys or Gln residue at the Xaa position, chemical modification of the ELP is possible. For example, McHale et al. used the enzyme transglutaminase to couple between Lys and Gln side chains in ELPs under mild conditions [11]. Chondrocytes were cultured for more than four weeks in the resultant hydrogel. Cell-compatible ELP hydrogels have also been obtained using click chemistry reactions [12,13]. The Lys residues of one ELP were modified with azide groups while those in the other ELP were functionalized with cyclooctyne groups. Catalyst-free click reactions occured on mixing these ELPs, yielding a hydrogel. The click hydrogels, with cell-adhesive and biodegradable properties, were successful in tissue regeneration, in animal models of volumetric muscle loss [14], myocardial infarction [15], and critical limb ischemia [16].

One of the characteristics of ELPs is the temperature-responsive self-aggregation properties. ELPs are soluble in cold water but become insoluble and coacervate above their transition temperature (*T*_t_), which can be controlled by adjusting the guest residue composition, molecular weight, and concentration of ELP [10,17]. Physically crosslinked ELP hydrogels, where the driving force of crosslinking is hydrophobic interactions, have been constructed by taking advantage of these temperature-responsive properties. While typical ELPs with XPGVG repeat sequence phase-separate from the solution above their *T*_t_, the substitution of the third Gly with Ala in the pentapeptide results in the formation of a hydrogel as a continuous phase [18]. This substitution slowed the ELPs’ dynamics above *T*_t_, enabling the formation of kinetically arrested and phase-separated nanostructures. Physically crosslinked hydrogels were also formed from the modular ELP consisting of alternating (VPGXG)*_n_* and polyalanine helix domains [19,20], amphiphilic ELP block copolymers with different temperature responses between the two blocks [21,22]. ELP motifs serve as a temperature-responsive building unit of fusion proteins. ELPs were combined with other self-assembling peptide units such as coiled-coil [23,24] and silk-like motifs [25,26,27] to give hydrogels formed by dual self-assembling mechanisms. These physically crosslinked hydrogels often have nonlinear viscoelastic properties, which are useful in cell encapsulation, hydrogel injection, and 3D bioprinting [28].

We have reported that double-hydrophobic ELP block copolymers with (VGGVG)_5_–(VPGXG)*_n_*–(VGGVG)_5_ (*n* = 25 or 50) sequences self-assembled to form well-defined nanofibers in aqueous solution [29,30]. Dynamic rheology measurements revealed that nanofiber dispersion, even at extremely low nanofiber concentration (0.034 wt%), exhibited solid-like behavior with storage modulus G′ greater than the loss modulus G″ over a wide range of angular frequencies [30]. This gel-like behavior is attributed to the formation of high-aspect-ratio (>500) nanofibers, through which percolated networks are formed in the solution. However, the gel fell under gravity and was not self-supportive. The objective of this research was to construct self-supporting hydrogels using double-hydrophobic ELPs at higher concentrations and to determine their rheological properties. We found that these ELPs, with or without a cell-binding sequence, can form self-supporting hydrogels in microtubes at 0.5 wt% polypeptide concentration. This concentration is an order of magnitude lower than those for previously reported ELP hydrogels (typically >5 wt%). Thixotropicity was revealed for the present hydrogels and the degree of self-recovery improved after chemical crosslinking of the nanofibers when intermolecular crosslinking was successful.

## 2. Results

### 2.1. Formation of Hydrogels

**Figure 1:**
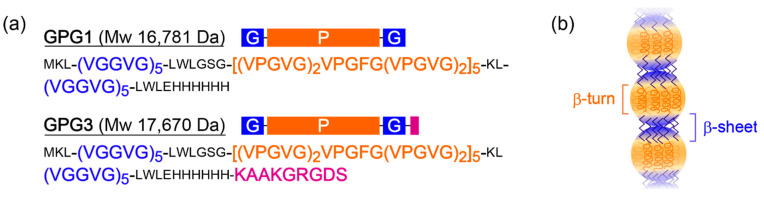
(**a**) Amino acid sequences of GPG1 and GPG3. (**b**) Plausible structural model for nanofibers of GPGs.

In this study, we used double-hydrophobic ELPs, GPG1 and GPG3 to obtain hydrogels (Figure 1). GPG1 and GPG3 contain a common self-assembling sequence (VGGVG)_5_–(VPGXG)_25_–(VGGVG)_5_, where X is V (80%) or F (20%) (F: Phe) to tune the *T*_t_ around room temperature [31]. The VPGXG motif in the central block and the VGGVG motif in the end blocks are derived from those in tropoelastin’s proline-rich and glycine-rich hydrophobic domains, respectively [32]. Only GPG3 has an additional KAAKGRGDS sequence (K: Lys, A: Ala, R: Arg, D: Asp, and S: Ser) at the C-terminus, in which KAAK is a motif from crosslinking domain of tropoelastin, and GRGDS is a cell-binding motif of fibronectin.

The mechanisms of nanofiber formation of GPGs have been proposed as follows [29,30,33]. They consist of a two-step pathway: (1) GPGs hydrophobically assemble to form nanoparticles rich in β-turn structures through dehydration of the (VPGXG)_25_ block, and (2) the nanoparticles connect into nanofibers along with the formation of β-sheet structures between the nanoparticles via (VGGVG)_5_ blocks (Figure 1b) [29,30,33].

**Figure 2:**
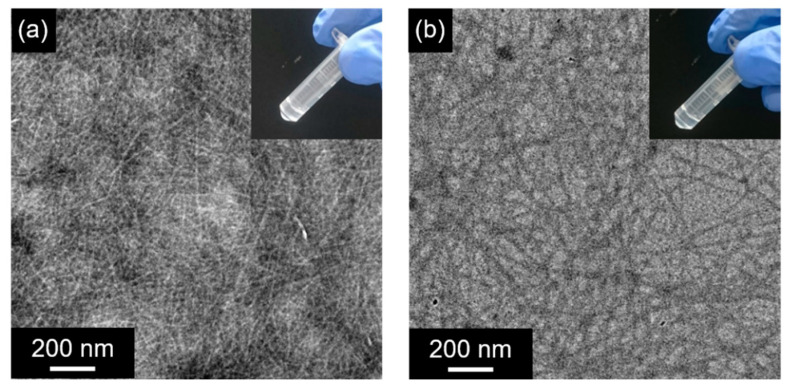
TEM images of the hydrogel of (**a**) 0.5 wt% GPG1, 1 d at 37 °C, (**b**) 0.5 wt% of GPG3, 7 d at 37 °C. Insets show photographs of the hydrogels.

GPG1 and GPG3 were each dissolved at 0.5 wt% in 10 w/v% sucrose aqueous solution followed by heating at 37 °C. The formation of self-supporting hydrogels in microtubes was confirmed after one day (1 d) for the sample containing GPG1 (Figure 2a inset). The sample that contains GPG3 remains fluidic after 1 d but lost the fluidity after 7 d (Figure 2b inset). These hydrogels were optically clear. Transmission electron microscopy (TEM) observation revealed the formation of nanofibers in these hydrogels (Figure 2a,b). The nanofibers are relatively straight with diameters of less than 10 nm.

### 2.2. Dynamic Rheological Characteristics of Hydrogels

**Figure 3:**
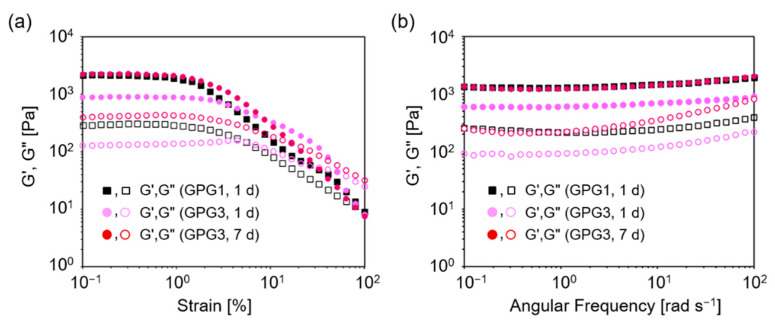
Rheological properties of hydrogels containing GPG1 and GPG3 at 0.5 wt%. (**a**) Strain sweep (1 Hz) and (**b**) frequency sweep (1% strain). The solid and open symbols represent G′ and G″, respectively.

Dynamic rheological characteristics of the hydrogels are shown in Figure 3. Strain sweep measurements of a GPG1 hydrogel formed after 1 d at 37 °C showed that G′ and G″ were relatively constant between 0.1% and 1% strains and both moduli decreased at above 2% strain (Figure 3a). This indicates a transition from a linear to nonlinear viscoelastic regime. Frequency sweep measurement of GPG1 hydrogel at 1% strain showed that G′ was approximately an order of magnitude higher than G″ for all ranges of frequencies measured (Figure 3b). In addition, G′ and G″ were constant regardless of the frequency. These characteristics highlight the elastic solid nature of the GPG1 hydrogel. Moduli for GPG3 hydrogels in the strain sweep measurements showed that gels formed after both 1 d and 7 d possess elastic properties up to 1% strain (Figure 3a). However, GPG3 gel at 1 d exhibited a characteristic trend in which G″ first increased and then decreased with increasing strain amplitude. This response is known as weak-strain overshoot [34] and was observed in some polymer solution systems [34,35] and peptide hydrogels [36]. In frequency sweep measurements, an increase in the loss moduli G″ at higher frequency was observed for GPG3 gel at 1 d and 7 d, indicating the contribution of fluid-like property (Figure 3b). The storage modulus G′ is related to the stiffness of the viscoelastic material, and the order of G′ values here is GPG1 (1 d) = GPG3 (7 d) > GPG3 (1 d).

**Figure 4:**
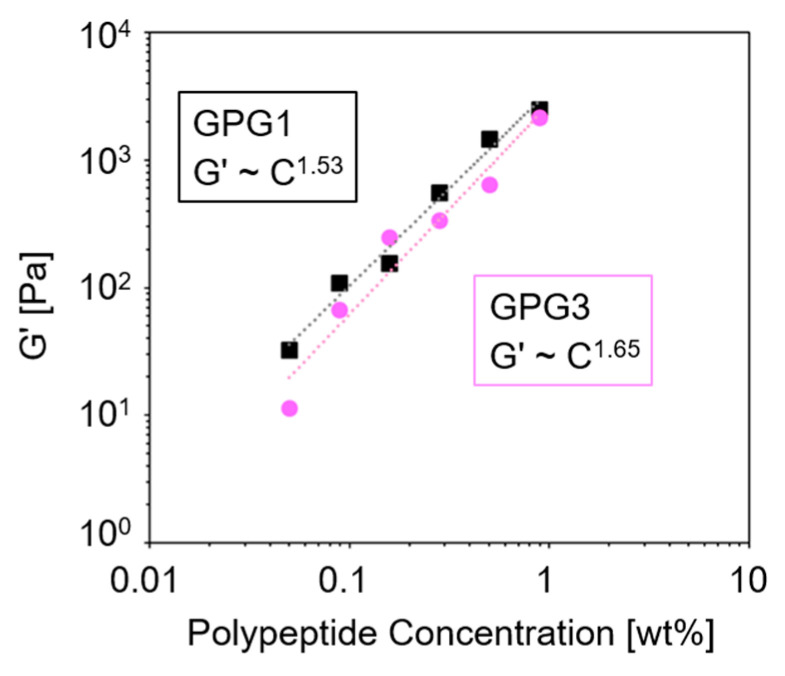
Storage modulus of GPG1 and GPG3 gels with various polypeptide concentrations (0.050 wt%–0.889 wt%) formed after 1 d at 37 °C. Time sweep measurements (1 Hz, 1% strain) were performed for each sample after being transferred to the sample stage, and the G′ values at 3600 s were plotted. The dashed lines represent fits to the data points; the equation of the line for GPG1: G′ = 3.48 × 10^3^C^1.53^, *R*^2^ = 0.984, and for GPG3: G′ = 2.76 × 10^3^ C^1.65^, *R*^2^ = 0.947).

Figure 4 shows the G′ of GPG1 and GPG3 hydrogels at different polypeptide concentrations. There is a power law between the storage modulus and the concentration (G′~C*^n^*) and the exponent *n* are 1.53 and 1.65 for GPG1 and GPG3 gels, respectively. The *n* is indicative of the network type [37]. For chemically crosslinked polymer networks, *n* is around 2.5 [38,39]. Values of *n* around 1.5 were reported for entangled semiflexible networks such as *F*-actin [40], unligated fibrin clot [41], filamentous bacteriophage [42], and nanofibers of synthetic peptides [36,37]. Therefore, it was suggested that the network structures obtained from GPGs are reminiscent of biological nanofiber systems.

### 2.3. Thixotropicity of Hydrogels

**Figure 5:**
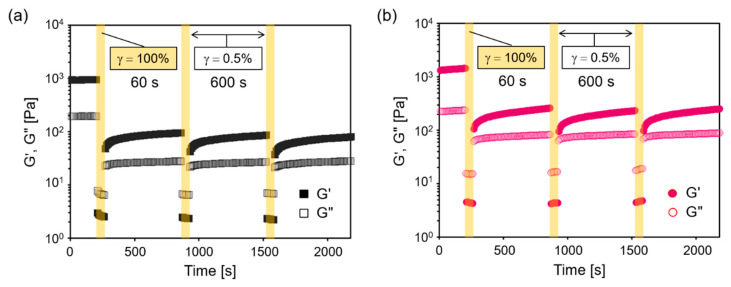
Changes of G′ and G″ in step-strain sweep dynamic rheological measurements (1 Hz). (**a**) 0.5 wt% GPG1, 1 d, (**b**) 0.5 wt% GPG3, 7 d.

One of the most interesting properties of the present hydrogels was their thixotropic (i.e., self-healing) nature, where the reversible breaking of the gel under mechanical strain, and the recovery at static conditions took place. The thixotropicity was confirmed with step-strain sweep rheological measurements consisting of the alternating application of high strain (*γ* = 100%, 60 s) and low strain (*γ* = 0.5%, 600 s) (Figure 5). In both GPG1 and GPG3 gels, G′ decreased immediately and became smaller than G″ when the high strain was applied. Upon returning to the low strain, G′ increased, and the relationships between G′ and G″ were reversed. These behaviors indicate the shear-induced, reversible gel–sol transition. Although G′ recovered instantaneously when high shear was removed, the following recovery was gradual and G′ did not completely recover to the initial values in the period of 600 s. The pattern of recovery was almost the same after the second cycle. The degree of recovery, the G′ after third cycles compared with the initial G′, was 8% and 17% for GPG1 and GPG3 gels, respectively.

### 2.4. Effects of Chemical Crosslinks Using Genipin

**Figure 6:**
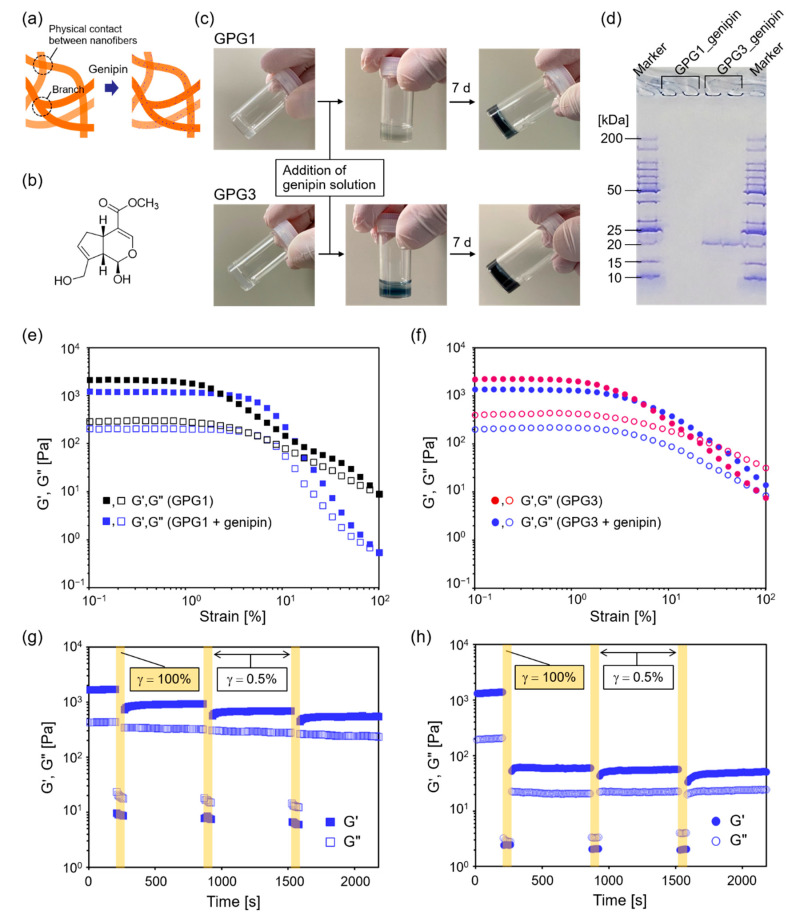
(**a**) Schematics of the chemical crosslinking of GPG nanofibers. (**b**) Chemical structure of genipin. (**c**) Change in the appearance of hydrogels by the addition of genipin solution. The supernatant solutions were removed in the photographs at 7 d. (**d**) SDS–PAGE of GPG1 and GPG3 gels after reacting with genipin for 7 d. (**e**,**f**) Effects of genipin crosslinking on the rheological properties of hydrogels containing (**e**) GPG1 and (**f**) GPG3 nanofibers at 0.5 wt% as a function of strain. The moduli of GPG1 gel at 1 d and GPG3 gel at 7 d in Figure 3a are re-plotted as references. (**g**,**h**) step-strain sweep measurements of (**g**) GPG1 and (**h**) GPG3 gels after genipin treatment.

We expected that the hydrogels would show improved self-healing ability when GPG nanofibers were chemically crosslinked because the permanent network structure will have resistance to the plastic deformation (Figure 6a). Genipin, a naturally occurring crosslinking agent with low cytotoxicity, was used for this purpose (Figure 6b) [43]. This compound forms chemical crosslinks between primary amino groups. The originally colorless genipin turns blue after the reaction. GPGs have primary amines at K residues and the N-termini; therefore, the number of the amino groups in GPG1 and GPG3 are three and five, respectively (Figure 1a). Because some reports suggest the dimerization of genipin molecules in the crosslinking reaction [44], the minimum required amount of genipin for complete crosslinking is the same as the number of amino groups in GPG molecules. Therefore, we used 1 and 2 mM genipin solutions, the amounts of genipin molecules which exceed those of amines of GPG1 and GPG3 gels, respectively. As shown in Figure 6c, gels of GPGs turned blue upon the addition of genipin solutions, and the color became stronger in 7 d. To estimate the degree of crosslinking, analysis using sodium dodecyl sulfate–polyacrylamide gel electrophoresis (SDS–PAGE) was performed (Figure 6d). GPG1 nanofibers after genipin treatment and SDS denaturation remained at the well of PAGE gel, indicating the formation of polymerized GPG1 that did not migrate in the PAGE gel. No monomer of GPG1 (*Mw* = 16,781 Da) was detectable. On the other hand, there was a band at around 20 kDa for GPG3 besides remaining proteins in the well. This result suggests that the monomer GPG3 (*Mw* = 17,670) or those decorated with genipin molecules were still present after genipin treatment.

Dynamic rheological measurements revealed that the range of linear viscoelastic regimes expanded to 3% strain for GPG1 gel after genipin treatment (Figure 6e). The G′ at the plateau region decreased from about 2- to 1-kPa after genipin treatment but still stayed in the same order. Moduli at 100% strain of genipin-treated gel were smaller than those of untreated gel. Different from GPG1, the linear viscoelastic region as a function of strain was unchanged for GPG3 gel after genipin treatment. However, there was no crossover of G′ and G″ after genipin treatment, while G′ = G″ at about 20% strain in unmodified GPG3 gel. This action indicates that GPG3 gel became more solid-like after the crosslinking reaction. The G′ values at the plateau region became smaller after crosslinking. Step-strain experiments demonstrated that the self-healing property of GPG1 gel improved (Figure 6g) compared to that without crosslinking (Figure 5a). The degree of recovery of G′ after the third cycle was ~32%. In contrast, the degree of recovery decreased to 3% for GPG3 after genipin treatment (Figure 6h).

## 3. Discussion

One of the requirements for hydrogels used in tissue regeneration and tissue model construction is that the biological and mechanical properties are independently controlled to discriminate these effects on tissue regeneration [45]. Therefore, we examined the dynamic viscoelastic properties of hydrogels of GPG1 and GPG3, in which only GPG3 has a cell-binding sequence, to find the condition in which GPG1 and GPG3 gels show similar stiffness. In our previous study, fibroblast cells attached and proliferated better on GPG3 nanofibers than on GPG1 nanofibers in 2D cell culture systems [31].

GPG1 (0.5 wt%) formed a self-supporting hydrogel in a microtube from 10 w/v% sucrose aqueous solution after 1 d at 37 °C while it took 7 d for GPG3, indicating the slower kinetics of nanofiber formation of GPG3 compared to GPG1. The weak-strain overshoot observed in the strain sweep rheological measurement of GPG3 gel at 1 d indicates that GPG3 is in the process of nanofiber formation, where both monomers and nanofibers of GPG3 are present (Figure 3a). We previously studied the process of nanofiber formation of GPG1 and GPG3 in ultrapure water at more dilute polypeptide conditions (20 μM), which correspond to 0.033 wt% and 0.035 wt% for GPG1 and GPG3, respectively [31]. Although the polypeptide concentration and solvent were different from this study, the slower assembly of GPG3 was also observed as indicated by the slower formation of β-sheet structures [31]. Although the difference in amino acid sequences between GPG1 (196 amino acids) and GPG3 (205 amino acids) is less than 5%, the highly charged KAAKGRGDS additional sequence exerts effects on the self-assembly kinetics probably because it provides electrostatic repulsion between molecules.

Notably, G′ values are similar between GPG1 gel formed at 1 d and GPG3 gel at 7 d in the frequency sweep dynamic rheological measurements (Figure 3b). This fact shows that these gels have similar stiffness but may have different biological activity, the properties of which are important for future cell study, because one can discuss the effect of only bioactive sequence while keeping the gel stiffness constant. However, we noted that G″ of GPG3 is more frequency-dependent, showing the contribution of liquid-like property. This phenomenon may be because the nanofiber formation of GPG3 does not reach equilibrium in 7 d.

To the best of our knowledge, this is the first report on the thixotropic behavior of ELP-based hydrogels. Gels containing GPG1 and GPG3 nanofibers immediately become sols on the application of high shear strain. G′ recovers to 8% and 17% of the initial G′ for GPG1 and GPG3, respectively, after three cycles of high and low strain applications. Although the mechanisms are not fully understood, similar self-healing behavior was observed in other peptide hydrogel systems [46,47,48,49,50]. Wychowaniec et al. pointed out that the recovery of G′ consisted of two processes with two characteristic times: a fast recovery process and a slower recovery process. The former process is likely a fast percolation of the globular hydrogel domains, which were clusters of original hydrogel generated under shear [46]. This accounts for the fast initial recovery of G′. The latter process contains further structural rearrangements of nanofibers that occur over time, leading to a slow increase in G′.

We hypothesized that if the plastic deformation under shear strain was minimized, then efficient recovery of G′ occurs because the shear-induced clusters retained the original nanostructures that could act as “nuclei” for the structural recovery [51]. Based on this idea, the stabilization of the physical network of GPG nanofibers was performed using genipin as a low-toxic chemical crosslinker (Figure 6). GPG1 hydrogels became more resistant to plastic deformation as shown by the increase in the linear viscoelastic regime as a function of strain (Figure 6e). The self-healing property of GPG1 is also improved after genipin crosslinking (Figure 6g). Note that G′ and G″ values at 100% strain after genipin treatment are higher than those without chemical crosslinking (Figure 5a), showing that our hypothesis is reasonable. It needs to be taken into account, however, that moduli at 100% strain of genipin-treated gel were smaller than those of untreated gel in the strain sweep measurements (Figure 6d). These results indicate that the process leading to high strain is one of parameters that affects gel disruption. Alternatively, genipin treatment rather resulted in the impaired self-healing ability of GPG3 gel (Figure 6h). We speculate that some of the genipins were used for intramolecular crosslinking, but not intermolecular crosslinking—genipins crosslinked two amino groups in the KAAK motif of GPG3. Intramolecular crosslinking was suggested from the results of SDS–PAGE (Figure 6d), where the band for GPG3 appeared at a higher position than that of the original GPG3 (17,670 Da) [31]. The modification of GPG3 by genipin might destabilize the self-assembled structure and make the nanofiber prone to dissociate at high strain, which caused a low extent of structural recovery.

## 4. Materials and Methods

### 4.1. Sample Preparation

GPG1 and GPG3 were expressed and purified as previously reported [31]. They were stored as lyophilized powders at −20 °C until use. To prepare hydrogels, polypeptide powders were dissolved in 10 w/v% sucrose (Wako Pure Chemical Industries, Osaka, Japan) aqueous solutions at 4 °C. Concentrations of the polypeptides were determined by measuring the absorbance at 280 nm using a Nanodrop 2000 UV-visible spectrometer (Thermo Fisher Scientific, Wilmington, DE, USA). Adequate amounts of 10 w/v% sucrose aqueous solutions were added to adjust polypeptides’ concentrations to 0.050 wt%, 0.089 wt%, 0.158 wt%, 0.281 wt%, 0.500 wt%, and 0.889 wt%. They were then incubated for 1 d (GPG1) and up to 7 d (GPG3) at 37 °C.

### 4.2. Rheological Measurements

The rheological measurements were conducted using an Anton Paar MCR302 rheometer with a 1° cone-and-plate configuration (25 mm diameter) and a 0.048 mm gap. The experiments which were controlled using an integrated Peltier system were performed at 37 °C. A solvent trap cover was used to keep the sample hydrated. The sample was transferred to the stage using a micropipette for fluidic dispersion or a spatula for hydrogels. They were left to equilibrate for 1 h on the stage at 37 °C before measurements.

### 4.3. TEM

TEM was performed using JEOL JEM-2100plus at an accelerating voltage of 200 kV. The samples were applied to a collodion-coated grid (Cu 100 mesh), stained with potassium Eu-encapsulated Preyssler-type phosphotungstate (Fujifilm Wako Pure Chemical Corporation, Osaka, Japan), and the excess solution was wiped away using blotting paper.

### 4.4. Genipin Treatment

Hydrogels of GPG1 and GPG3 with 0.5 wt% polypeptide concentration were obtained after incubation at 37 °C for 1 d and 7 d, respectively. Genipin (Wako Pure Chemical Industries, Osaka, Japan) was dissolved in 10 w/v% sucrose aqueous solution to final concentrations of 1 mM and 2 mM. The genipin solution (1 mM or 2 mM) was carefully added to the top of the GPG1 or GPG3 gel, in which the volume of genipin solution and the hydrogel was 1:1. They were then incubated at 37 °C for one day, followed by the removal of supernatants, and further incubated at 37 °C for an additional six days. These genipin-treated gels were subjected to SDS–PAGE analysis. The gels (18 μL) were denatured by mixing sample buffers (6 μL) containing SDS (Wako Pure Chemical Industries, Osaka, Japan) followed by incubation at 37 °C for 30 min. They were applied to the wells of a PAGE precast gel (e-PAGEL E-T 12.5 L, Atto Corporation, Tokyo, Japan) and electrophoresed at a constant current of 35 mA.

## 5. Conclusions

In this paper, the formation of hydrogels composed of self-assembled nanofibers of double-hydrophobic elastin-like block polypeptides, GPG1 and GPG3, is demonstrated for the first time. The hydrogels are obtained at the low polypeptide concentation of 0.5 wt%. GPG3 having a cell-adhesion sequence has slower kinetics in hydrogel formation than GPG1, while the storage moduli G′ of mature gels are similar between GPG1 and GPG3 gels. The power law between G′ and polypeptide concentration suggests that GPG gels consist of entangled semiflexible networks as found in biological gel systems. The GPG gels show thixotropic behavior in which reversible gel–sol transition occurs with low and high shear strain applications. The degree of recovery of G′ upon removal of high strain is improved in GPG1 gel after genipin treatment, where intermolecular crosslinking is successful. In contrast, the recovery is hampered in GPG3 gel after genipin treatment, probably because of the intramolecular crosslinks that interfere with the self-assembly of GPG3 molecules. These findings would provide deeper insight into the structure–property relationships of the self-assembling polypeptides [52] and a better design strategy for hydrogels with desired viscoelastic properties.

## Data Availability

Not applicable.
